# Annotation and expression of carboxylesterases in the silkworm, *Bombyx mori*

**DOI:** 10.1186/1471-2164-10-553

**Published:** 2009-11-24

**Authors:** Quan-You Yu, Cheng Lu, Wen-Le Li, Zhong-Huai Xiang, Ze Zhang

**Affiliations:** 1 The Institute of Agricultural and Life Sciences, Chongqing University, Chongqing 400044, China; 2 The Key Sericultural Laboratory of the Agricultural Ministry of China, Southwest University, Chongqing, 400716, China

## Abstract

**Background:**

Carboxylesterase is a multifunctional superfamily and ubiquitous in all living organisms, including animals, plants, insects, and microbes. It plays important roles in xenobiotic detoxification, and pheromone degradation, neurogenesis and regulating development. Previous studies mainly used Dipteran *Drosophila *and mosquitoes as model organisms to investigate the roles of the insect COEs in insecticide resistance. However, genome-wide characterization of COEs in phytophagous insects and comparative analysis remain to be performed.

**Results:**

Based on the newly assembled genome sequence, 76 putative COEs were identified in *Bombyx mori*. Relative to other Dipteran and Hymenopteran insects, alpha-esterases were significantly expanded in the silkworm. Genomics analysis suggested that *BmCOEs *showed chromosome preferable distribution and 55% of which were tandem arranged. Sixty-one *BmCOEs *were transcribed based on cDNA/ESTs and microarray data. Generally, most of the COEs showed tissue specific expressions and expression level between male and female did not display obvious differences. Three main patterns could be classified, i.e. midgut-, head and integument-, and silk gland-specific expressions. Midgut is the first barrier of xenobiotics peroral toxicity, in which COEs may be involved in eliminating secondary metabolites of mulberry leaves and contaminants of insecticides in diet. For head and integument-class, most of the members were homologous to odorant-degrading enzyme (ODE) and antennal esterase. RT-PCR verified that the ODE-like esterases were also highly expressed in larvae antenna and maxilla, and thus they may play important roles in degradation of plant volatiles or other xenobiotics.

**Conclusion:**

*B. mori *has the largest number of insect COE genes characterized to date. Comparative genomic analysis suggested that the gene expansion mainly occurred in silkworm alpha-esterases. Expression evidence indicated that the expanded genes were specifically expressed in midgut, integument and head, implying that these genes may have important roles in detoxifying secondary metabolites of mulberry leaves, contaminants in diet, and odorants. Our results provide some new insights into functions and evolutionary characteristics of COEs in phytophagous insects.

## Background

Carboxylesterase (COE, EC 3.1.1.1) is a multigene family and occurs in animals, plants, insects, and microbes [1-4]. COEs are mainly attributed to B esterases, which were essentially irreversibly inhibited by organophosphate insecticides (OPs). Based on sequence similarity and substrate specificity, insect COE genes can be subdivided into eight subfamilies: α-esterase (ae), β-esterase (be), juvenile hormone esterase (jhe), gliotactins (gli), acetylcholinesterases (ace, AChE), neurotactins (nrt), neuroligins (nlg), and glutactin (glt) class [3]. α-esterases, β-esterases, acetylcholinesterases and juvenile hormone esterase account for the majority of the catalytically active COEs [3]. Gliotactins, neurotactins, neuroligins, and glutactin classes are generally considered to be noncatalytic but have a variety of functions essential to development and neurogenesis [5].

COEs have a broad range of functions; the key role is hydrolyzing esters of carboxylic acids. Carboxylesterases are also a class of the metabolic enzymes involved in insecticide resistance, which are implicated in the resistance of insects to OPs, carbamates, and pyrethroids through gene amplification, upregulation and coding sequence mutations [6]. Furthermore, COEs also play important roles in allelochemical metabolism and tolerance, although the roles were validated only at the biochemical level in a few cases [6]. In addition, carboxylesterases can serve as noncatalytic adhesive proteins involved in cell-to-cell interactions [5] and participate in other functions, such as pheromone degradation in moths [7] and hydrolysis of the neurotransmitter acetylcholine and juvenile hormone (JH) [8,9].

Studies on insect carboxylesterases have been mainly focused on mediating insecticide resistance [6,10]. Relatively, the mechanism of degrading plant allelochemicals is still unclear, and only some biochemical evidence confirmed that COEs were related to detoxification of the secondary metabolites of plants. Carboxylesterases can be induced by phenolic glycosides in *Papilio Canadensis *[11], and its activity was positively correlated with the survival rate of the gypsy moth, suggesting that esterase may be responsible for glycoside metabolism [12]. In the tobacco cutworm, *Spodoptera litura*, sublethal doses of the widely occurring plant glycoside rutin resulted in a significant increase in midgut carboxylesterase activity [13]. It was also found that COEs can be induced by indole alkaloid gramine in *Sitobion avenae*, and the increase of COE activity was positively correlated with dietary gramine concentrations, suggesting that COEs were involved in gramine detoxification [14]. In addition, quercetin, rutin and 2-tridaconone can also induce the activities of COEs in insects [15,16].

Herbivorous animals encounter a wide variety of secondary products in the plants on which they feed. They must therefore have developed mechanisms to metabolically inactivate some of the potentially toxic plant chemicals that they ingest. Silkworm is phytophagous insect, and specifically feeds on mulberry, which also encounters a mass of allelochemicals from its host plant. Because the silkworm grows well on mulberry leaves, the toxicities and defensive activities of these leaves against herbivorous insects have been overlooked. However, a recent study revealed that mulberry latex rich in sugar-mimic alkaloids was highly toxic to caterpillars [17]. Some alkaloids contained in mulberry leaves are potential inhibitors of mammalian digestive glycosidases but not inhibitors of silkworm midgut glycosidases, suggesting that the silkworm has enzymes specially adapted to enable it to feed on mulberry leaves [18]. In addition, β-fructofuranosidase was characterized in the silkworm genome, which has been no direct experimental evidence that this gene is encoded in the genome of animals [19]. *Bmsuc1 *played an important role in avoiding the toxic effects of 1,4-dideoxy-1,4-imino-D-arabinitol (D-AB1) and 1-deoxynojirimycin (DNJ) that are present in extremely high concentrations in the mulberry latex. In the "animal-plant warfare", silkworm has developed the mechanisms to metabolically inactivate those potentially toxic chemicals, such as detoxification enzyme carboxylesterase, cytochrome P450 monooxygenases (P450) and glutathione S-transferase (GST), etc. Thus, silkworm can be used as a model of the insect-plant interaction.

*B. mori *is an economically important insect and the Lepidoptera model for the study of pest control in agriculture. Recently, the fine genome map of the silkworm has been assembled. Totally, 87% of the scaffold sequences were anchored to all 28 chromosomes and 14,623 genes were predicted [20]. In addition, carboxylesterases are a functionally important superfamily, which play important roles in insecticide resistance, allelochemical tolerance, and developmental regulation. Previous studies on silkworm carboxylesterases mainly focused on isozyme polymorphism [21-23]. Herein, we present the identification and genomic analysis of silkworm COEs using the newly assembled 9× genome sequence. We have searched available EST data for each silkworm COE to confirm active transcription and examined the expression patterns using the genome-wide microarray of the silkworm [24]. Studying the expressions and evolutionary aspects of such large family of COEs will help us understand its functional versatilities.

## Results and Discussion

### Annotation and phylogeny of *B. mori *COEs

*Drosophila melanogaster*, *Anopheles gambiae *and *Apis mellifera *COEs were retrieved from GenBank and used for blast search against the new assembly of the silkworm genome to characterize the COE superfamily in *B. mori*. Through genomic analysis and gene prediction, 76 putative COE genes were identified in the silkworm genome (Additional file 1). This indicated that the *B. mori *genome contained more COE members compared with *D. melanogaster *(35), *An. mellifera *(24), and *Ap. gambiae *(51) (Table 1).

**Table 1:** Comparison of the gene number for COEs in *B. mori*, *D. melanogaster, Ap. Mellifera *and *An. gambiae*

Class/clades	*B. mori*	*D. melanogaster*	*Ap. mellifera*	*An. gambiae*
intracellular catalytic class				
A clade, α-esterase	42	1	0	0
B clade, α-esterase	13	0	8	0
C clade, α-esterase	0	12	0	16
secreted catalytic class				
D clade, JHE	4	0	1	4
E clade, integument esterase	2	3	1	0
F clade, JHE	0	2	0	5
G clade, β-esterase	2	3	3	5
H clade, uncharacterized	1	1	1	1
I clade, glutactin	0	4	0	9
neurodevelopmental class				
J clade, AChE	2	1	2	2
K clade, uncharacterized	1	1	1	1
L clade, gliotactin	1	1	1	1
M clade, neuroligin	6	4	5	5
N clade, neurotactin	2	2	1	2
total	76	35	24	51

Note: Classification of *An. gambiae *COEs was based on Oakeshott et al. (2005) [5] and Claudianos et al. (2006) [25]. Genes in clade D for *An. gambiae *were the uncharacterized proteins.

**Figure 1:**
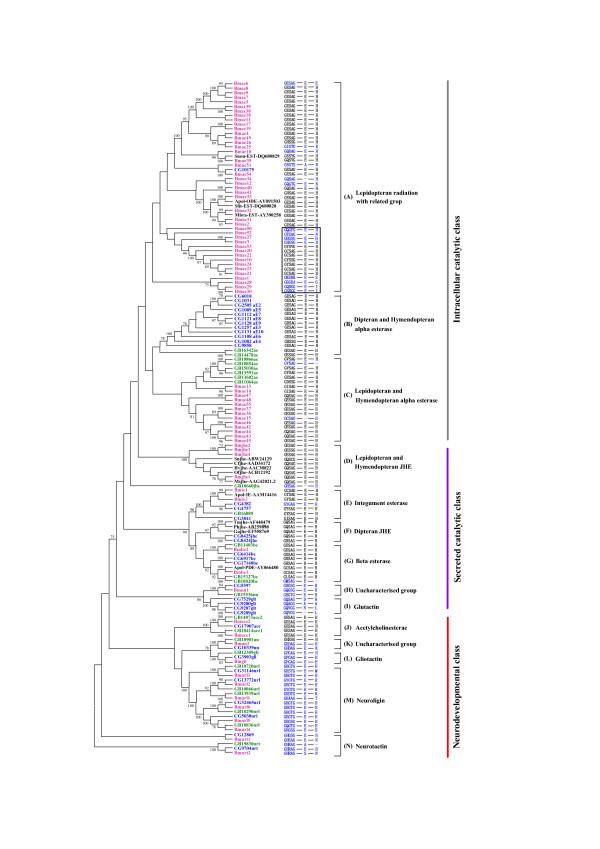
**Neighbor-joining tree of amino acid sequences from *B. mori*, *Ap. mellifera*, and *D. melanogaster*, and some known COEs in other Lepidopteran insects**. Bootstrap values > 70% are shown. The catalytic triad was predicted by blastp searching NCBI conserved domain database (CDD) http://www.ncbi.nlm.nih.gov/Structure/cdd/cdd.shtml using COE amino acid sequences as queries. Thus, the catalytic residues (S200, E327 and H440) and GXSXG consensus sequence around the active site serine were presented. Short dash: absence of the catalytic residue. Red: *B. mori*; Green: *Ap. mellifera*; Blue: *D. melanogaster*; Black: other Lepidoptera. *Snon *(*Sesamia nonagrioides*), *Apol *(*Antheraea polyphemus*), *Slit *(*Spodoptera littoralis*), *Mbra *(*Mamestra brassicae*), *Sn *(*Sesamia nonagrioides*), *Cf *(*Choristoneura fumiferana*), *Hv *(*Heliothis virescens*), *Of *(*Omphisa fuscidentalis*), *Ms *(*Manduca sexta*), *Tm *(*Tenebrio molitor*), *Ph *(*Psacothea hilaris*), *Ga *(*Gryllus assimilis*).

The neighbor-joining tree of COEs in *B. mori*, *D. melanogaster*, *Ap. mellifera*, and some related species was reconstructed (Figure 1). It can be seen from Figure 1 that the topology of phylogenetic tree was very similar to those obtained in previous studies [5]. Insect carboxylesterases can be divided into fourteen clades and three major classes (intracellular catalytic, secreted catalytic, and neurodevelopmental classes) based on the phylogenetic tree. The gene numbers of neurodevelopmental class and secreted catalytic class were alike in the four organisms, especially, the orthologous genes in neurodevelopmental class can be unambiguously defined (Figure 1, Table 1). Thus, this class of COEs might be involved in essential steps in conserved physiological pathways and subject to function constraints. While α-esterases were independently expanded in *D. melanogaster*, *Ap. mellifera*, and *An. gambiae*, the silkworm α-esterases experienced an obvious species-specific expansion: 55 α-esterase members were identified. This suggested that this class of COEs may play important roles in the adaptation of these insects to their specific biological niches rather than fulfilling general housekeeping functions.

### Expansion of intracellular catalytic class in the silkworm

The intracellular catalytic class of COEs belongs to α-esterases, which function to detoxify xenobiotics and some members are related to organophosphorus insecticides (OPs) resistance in insects [5,25,26]. This class (clades A-C) includes 55, 16, 13, and 8 esterases in *B. mori*, *An. gambiae*, *D. melanogaster *and *Ap. mellifera*, respectively (Figure 1, Table 1). Intracellular catalytic esterases in the silkworm were in clades A and C, while the corresponding esterases in *D. melanogaster *and *Ap. mellifera *were mainly located in the clades B and C, respectively. All the other sequences in clade A came from Lepidopteran insects except for the *D. melanogaster CG10175 *that shared 37.7% amino acid identity with *Bmae54*. Furthermore, most of the silkworm α-esterases were located in this clade.

Intracellular catalytic carboxylesterases have some common characteristics, such as conserved catalytic triad S200, E327 and H440, the numbering of which is that of *torpedo californica *AChE [27]. The catalytic triad of COEs was predicted by blastp searching NCBI conserved domain database (CDD) using COE amino acid sequences as queries (Figure 1). The results indicated that most of the intracellular catalytic carboxylesterases in the silkworm had GESAG consensus sequences similar to *Drosophila*. However, 15 of 55 silkworm α-esterases changed one or more residues of the catalytic triad. Most of the variants of the catalytic triad in the silkworm α-esterases were phylogenetically related to the variants of GESAG, which formed a cluster boxed on the phylogenetic tree. Similarity analysis of amino acid sequences indicated that α-esterases with substitutions in catalytic triad shared < 30% identity with other α-esterases, and most of them were only about 20%. These results indicated that those rapidly evolved α-esterases in the silkworm might lose their hydrolyzing functions.

**Figure 2:**
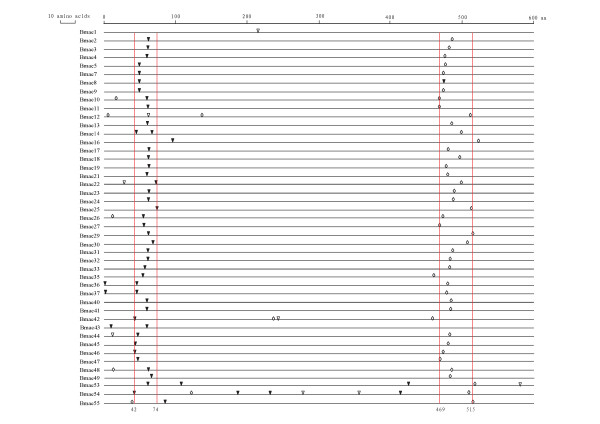
**Location of introns of the silkworm α-esterases**. Inverted black triangle: phase 0; Inverted white triangle: phase 1; White diamond: phase 2. Only those COE genes with putative complete coding sequences are shown. The vertical lines were the boundary of conservative distribution of introns.

The intron number and location of silkworm α-esterase were analyzed. In total, 111 introns were found in 45 α-esterases with putative complete coding sequence (Figure 2). For intron number, most of the α-esterases (30 out of 45) contained only two introns. While *Bmae1 *had only one, others contained three or more introns, especially, *Bmae54 *contained eight introns. The intron locations were relatively conserved for the α-esterases. The first conserved one was located between the 42th and 74th amino acids whereas the second one lay between the 469th and 515th amino acids. In addition, almost all of the first and second introns were phase 0 and phase 2, respectively. Thus, the gene structure of the silkworm α-esterases was relatively conserved.

Lepidopteran odorant-degrading enzyme (ODE) and antennal esterase play an important role in inactivating pheromone because degradation of odorant molecules is a necessity to avoid the continuous stimulation of the receptors [28,29]. Those known ODEs and antennal esterases of Lepidopteran insects were also included in the phylogenetic analysis. The results indicated that some of the odorant-degrading esterase were clustered with silkworm α-esterases in clade A. Silkworm *Bmae33*, *Antheraea polyphemus *ODE (*Apol*-ODE) and *Spodoptera littoralis *ODE (*Slit*-EST) might be orthologous genes, and *Bmae33 *shared 73.1% of amino acid identity with *Apol*-ODE and 64.6% with *Slit*-EST. *Bmae35 *was phylogenetically closely related to *Sesamia nonagrioides *ODE (*Snon*-EST); they shared 48.6% identity. In addition, *Bmae32 *was homologous with *Mamestra brassicae *antennal-specific esterase (*Mbra*-EST); they had 58.8% identity. Thus, existence of putative ODEs in Lepidoptera was one of the reasons that α-esterases were obviously expanded in the silkworm compared with Dipteran and Hymendopteran insects.

### Secreted catalytic class

Juvenile hormone esterase (JHE), integument esterase, β-esterase, and glutactin belong to the secreted catalytic class. In this class, uncharacterized esterases were also identified. JHEs play important roles in development, metamorphosis, diapause and reproduction in insects, which can hydrolyze and regulate the titre of juvenile hormone (JH) [8]. Four putative JHEs were identified in the silkworm genome (Figure 1). Silkworm JHEs showed a moderate expansion compared with *D. melanogaster *(2), *Ap. mellifera *(1), *An. gambiae *(5) [5,30], and *Aedes aegypti *(10) [31]. The phylogenetic tree indicated that Lepidopteran and *Ap. mellifera *JHEs were clustered together, while Dipteran and Coleopteran JHEs formed another cluster. Thus, this provides support for the hypothesis that there are two separate origins of JHE in the insect esterases [30].

*Bm*JHE1 was phylogenetically related to *Cf*JHE, *Hv*JHE, *Of*JHE and *Ms*JHE, and shared higher identities with them (49.6% - 60.8%). Thus, they might be orthologous genes (Figure 1). However, *Bm*JHE1 and the other three *Bm*JHEs showed lower identities (42.4% - 44%) than those among *Bm*JHE2, *Bm*JHE3 and *Bm*JHE4 (72.0% - 74.6%). So, we predicted that *Bmjhe2*, *jhe3 *and *jhe4 *were recently duplicated by *Bmjhe1*. JHEs contain some specific characteristics, such as a GQSAG core catalytic motif required for JH-specific esterase activity [5], a particular amphipathic helix as for a characteristic of Lepidopteran JHEs [32]. The analysis of catalytic triad indicated that *Bm*JHE1 contained the specific GQSAG motif while *Bm*JHE2 and *Bm*JHE4 had the GESAG, and *Bm*JHE3 had GESSG. Only *Bm*JHE1 and *Bm*JHE4 were identified to have the three Args (R 174, 181, 185) along one face of an amphipathic helix. In addition, *Bm*JHE1 has been verified to have JH-specific esterase activity *in vivo *[33,34]. Based on sequence characteristics and known functional data, we speculated that *Bm*JHE1 was the only one physiologically functional JHE in the silkworm, and that those new duplicated JHEs might have evolved other functions.

In insects, most of pheromone molecules are strongly hydrophobic and therefore tend to adhere to waxy surfaces. Both male and female may need to remove the pheromones from their integument so that they can better identify and control the signal, respectively [35]. During this signaling, integument esterase plays important role in inactivation of pheromones [29,35]. In total, two putative integument esterases were identified in the silkworm (Figure 1). One and three integument esterases were identified in *Ap. mellifera *and *D. melanogaster*, respectively, whereas none was found in *An. gambiae *(Table 1). Previous study found that *Apol*-IE in *Ant. polyphemus *was distributed in adult antennae and legs, suggesting that it may have the function of degrading pheromone [29]. *Bmie1 *shared 62.6% identity with *Apol*-IE at amino acid level; they were phylogenetically related and may be orthologous genes. In addition, identities among silkworm and *Drosophila *integument esterases were about 44%. Thus, integument esterases among insect species showed higher similarities and played similar roles in degrading pheromone or detoxifying xenobiotics entered into integument.

Although the number of β-esterase genes is not big in insects, these enzymes have multiple functions, including metabolic resistance to OPs and carbamates in Hemipteran insects [5], reproductive function in Diptera [5], and pheromone signaling in Lepidoptera [36]. In total, two β-esterases were found in the silkworm, three in *Ap. mellifera *and *D. melanogaster *and five in *An. gambiae*. *Bmbe2 *shared 43.6% and 39.1% amino acid identities with GB11403 and CG6414, respectively, and they were clustered together on the phylogenetic tree and may be 1 : 1 : 1 orthologs (Figure 1). *Bmbe1 *is the ortholog of *Apol*-PDE (pheromone-degrading enzyme) in *Ant. polyphemus*, which plays important role in validated rapid inactivation of sex pheromone [36]. Thus, *Bmbe1 *may have similar function to *Apol*-PDE, involved in pheromone signaling.

Glutactin is a novel *Drosophila *basement membrane related glycoprotein located in the envelope of the developing nervous system; it may play a role in intercellular ordering and adhesion [37-39]. Four and nine glutactins were found in *D. melanogaster *and *An. gambiae*, respectively, while no glutactin gene was found in the silkworm and *Ap. mellifera*. However, uncharacterized clade (H) was phylogenetically related to *Drosophila *glutactin clade. *Bmun1 *shared 36.2% and 32.3% amino acid identities with CG5397 and GB15536, respectively, and the three genes may be 1 : 1 : 1 orthologs. Whether *Bmun1 *had the function of glutactin or how this function was substituted in the silkworm remains to be determined.

### Conserved neurodevelopmental class

In neurodevelopmental class, orthologs among insects can be easily identified (Figure 1). Thus, these genes were generated by duplication events occurred before insect radiation and might have experienced purifying selection process after speciation. Acetylcholinesterases (AChEs) were the only enzymes that perform catalytic function in the neurodevelopmental class. Furthermore, they may be the important target of OPs [30]. In Drosophilidae and Muscidae, only one ace gene was identified, while two ace genes were found in other Dipteran, Hymenoptera, and Lepidopteran insects. On the phylogenetic tree, ace clade can be obviously divided into ace1 and ace2 subclades (Figure 1). In the silkworm, *Bm*AChE1 and *Bm*AChE2 shared only 31.5% amino acid identity and 54.5% and 32.6% amino acid identities with *Am*AChE1, 31.9% and 60.0% with *Am*AChE2, 29.1% and 50.4% with *Dm*AChE2, respectively. However, either *Bm*AChE1 or *Bm*AChE2 showed higher conservation with other Lepidopteran insect AChE1 or AChE2 (Additional file 2). *Bm*AChE1 shared 72.9% - 98.6% amino acid identities with orthologous gene from *Bombyx mandarina*, *Helicoverpa assulta*, *Helicoverpa armigera*, *Cydia pomonella*, and *Plutella xylostella*, and *Bm*AChE2 showed the 91.2% - 99.2% identities with the corresponding orthologs from species above.

The major function of AChE is hydrolysis of the neurotransmitter acetylcholine bounded at cholinergic synapses in the central nervous system of insects [40], conferring target site resistance to OP and carbamat insecticides [5]. In silkworm, both *Bm*AChE1 and *Bm*AChE2 contained the catalytic triad and disulfide bridges (C^88^-C^115^, C^275^-C^286 ^and C^423^-C^542 ^in Torpedo AChE [40], Additional file 2). In addition, an inhibition assay indicated that both of them can be inhibited by eserine and paraoxon [41]. However, the alignment of known Lepidopteran AChEs indicated that AChE1 had protrudent C-terminal compared with AChE2, and AChE2 (position at 150-230 aa) had an insertion of 18 incontinuous amino acids like the hydrophilic insertion in DipteranAChE, which had 31 residues [42]. Thus, *Bm*AChE1 and *Bm*AChE2 showed obvious differentiation in sequence. Previous studies indicated that the AChE2 in those insects with two *ace *gene system had cell-to-cell communication/adhesive properties [5]. Thus, we speculated that *Bm*AChE2 could substitute the function of glutactin due to the loss of glutactin gene in the silkworm and that *Bm*AChE1 had the function of hydrolyzing acetylcholine.

Generally, gliotactin, neuroligin, and neurotactin are noncatalytic adhesive proteins involved in cell-to-cell interactions [5]. Single gliotactin gene was identified in the *B. mori *genome as *D. melanogaster *and *Ap. mellifera *(Figure 1), and these orthologous genes shared about 52% amino acid identities. Like *D. melanogaster*, two putative neurotactin genes were found in the silkworm. CG9704, GB19830 and *Bmnrt2 *were orthologous genes, which showed about 40% amino acid identities. Compared with gliotactin and neurotactin, neuroligin genes were obviously duplicated in the *B. mori*, *D. melanogaster *and *Ap. mellifera*, corresponding to 6, 4 and 5 duplicates, respectively. The neuroligin clade (M) contained three pairs of 1 : 1 : 1 orthologs. This indicated that most of the neuroligins were duplicated before radiation of the three species. In addition, except for the known families, uncharacterized group was also found in clade (M). *Bmun2*, CG10339 and GB18901 were 1 : 1 : 1 orthologous genes, and *Bmun2 *shared 40.1% and 40.9% identities with CG10339 and GB18901, respectively.

### Genomic distribution of *BmCOEs*

**Figure 3:**
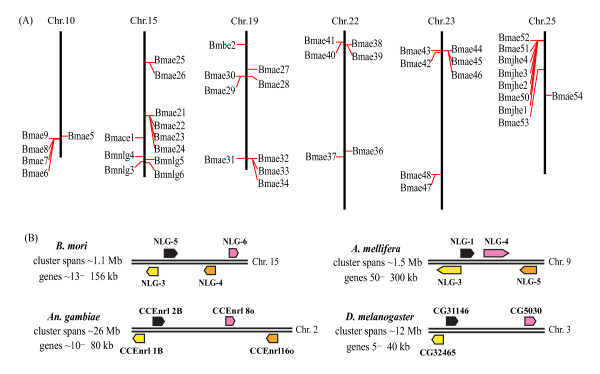
**The cluster organization of *BmCOEs *and microsynteny of neuroligins**. (A) The cluster organization of *BmCOEs *in the silkworm genome. Only those genes involved in clusters of five or more COE genes on the same chromosomes are shown. (B) microsynteny of neuroligins among *B. mori*, *D. melanogaster*, *An. gambiae*, and *Ap. Mellifera*. The infromation for *D. melanogaster*, *An. gambiae*, and *Ap. Mellifera *came from [25]. The arrows represent gene localization and transcriptional orientation.

In the fine genome map of the silkworm, most of the silkworm COEs could be mapped to chromosomes. Totally, 73 out of 76 silkworm COEs were distributed on 19 chromosomes and there are 13 *BmCOE *clusters on different chromosomes (Additional file 1 and 3). About 55% of *BmCOE *genes were tandem arranged in the silkworm genome. Moreover, *BmCOE *genes were not evenly distributed on chromosomes and about 62% COEs were massively located on six chromosomes (Figure 3a).

Generally, tandem arranged genes showed higher similarities one another and could be classified into a common family. For instance, *Bmae6 - 9 *were located on chromosome 10 and shared 89.1% - 95.7% amino acid identities. Although the *Bmae5 *is away from the gene cluster on the same chromosome, it showed high similarities (about 82% identity) to the genes in the cluster (Figure 3a). Most of the other tandem arranged COEs also showed 40% - 50% amino acid identities among members in a cluster. The tandem arranged COEs might be created by local duplications. Tandem arranged COEs tend to form a family, however, some are not the case. For example, *Bmnlg1 *and *Bmae2 *were tandem arranged but shared only 17% identity identities, and located in a mixed gene cluster with three alpha-esterases (*Bmae50 - 52*) and four JHEs (*Bmjhe1 - 4*). Thus, these tandem arranged COEs might be created by other mechanisms, not by local duplication or they are very old duplicates.

Neuroligins showed remarkable conservation of microsynteny among the *Ap. mellifera*, *D. melanogaster*, and *An. gambiae *genomes [25]. In the silkworm genome, six neuroligins were found; four of six were located on the chromosome 15, which spanned about 1.1 Mb (Figure 3b). The four silkworm neuroligins showed similar gene arrangement to those in *Ap. mellifera*, *D. melanogaster*, and *An. gambiae*. Thus, such microsynteny of neuroligins was present in these four organisms. The only difference is that the location of the NLG4 and NLG6 in *Ap. mellifera *and *An. gambia *was changed in *B. mori*.

### Expression profiles of *BmCOEs*

#### ESTs analysis

In order to detect the expression of the *B. mori *COEs, we searched the silkworm dbEST database downloaded from GenBank using the putative coding sequences as queries. The results indicated that 47 COEs matched at least one EST and most of the transcriptionally active genes were specifically expressed in tissues.

#### Microarray-based gene expression profiles in multiple tissues

**Figure 4:**
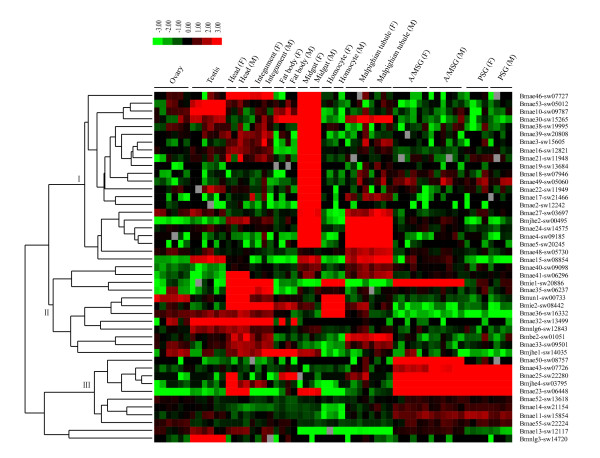
**Tissue expression patterns of the silkworm COEs in different larvae tissues based on microarray data**. Hierarchical clustering with the average linkage method was performed using Cluster software http://genome-www.stanford.edu/clustering/. F: female; M: male. A/MSG: anterior/median silk gland; PSG: posterior silk gland.

Based on the silkworm genome-wide microarray dataset http://silkworm.swu.edu.cn/microarray[24], expressions of *BmCOEs *in multiple tissues on the day three of the fifth instar were surveyed. It was found that 63 COE genes contained the oligonucleotide probes, whereas only 45 COEs showed expression signals, with signal value >400 at least in one tissue. The signal values of those expressed COEs were clustered to analyze the expression profiles (Figure 4). The results indicated that almost all of the COEs showed tissue specificity, including midgut-, head and integument-, and silk gland-specific expressions.

The catalytic triad is the foundation of carboxylesterases that perform catalytic function. For the *D. melanogaster *and *Ap. mellifera *α-esterases, only GB10854 gene lost the His of the catalytic triad. Fifteen α-esterases were found in the silkworm but they mutated in the catalytic triad, which may result in loss of the hydrolase activity. Based on the ESTs/microarray datasets, most of these α-esterases (11/15) had expression evidence (Additional file 4). Thus, these α-esterases may acquire new functions. Previous studies indicated that esterase gene amplification is one of the important mechanisms resistant to organophosphorus and carbamate insecticides [43]. The amplified esterases can produce broad spectrum insecticide resistance through rapid-binding (sequestration) mechanism. So we supposed that these amplified α-esterases may play roles in sequestering secondary metabolites of mulberry leaves or insecticide contaminants in diet. In addition, due to mutation of the catalytic triad and no expression evidence, *Bmae6 *and *Bmae28 *may be pseudogenes.

#### Midgut-specific expression genes

Twenty-two *BmCOE *genes were found to be predominantly expressed in the midgut and their expression levels did not significantly differ between male and female (Figure 4, group I). Except for *Bmjhe2*, all the midgut-specific genes belonged to α-esterases. It should be noted that *Bmae3*, *ae18*, *ae27*, and *ae30 *mutated in the catalytic triad were also included in this class. Thus, most of the midgut-specific expression genes should have hydrolyzing function. The potential hazard faced in sericulture is the occasional contamination of the mulberry leaves by air-borne insecticides that have been used in neighboring fields. Midgut is the first barrier of xenobiotics peroral toxicity, in which COEs can eliminate insecticides, such as OPs, carbamate insecticides, on mulberry leaves.

Silkworm is the phytophagous insect, and specifically feeds on mulberry, which also encounters a mass of allelochemicals from its host plant. A recent study revealed that mulberry latex rich in sugar-mimic alkaloids was highly toxic to caterpillars [17]. That silkworm is less affected by sugar-mimic alkaloid is due to insensitivity of *B. mori *glycosidases and β-fructofuranosidase [18,19]. So, during the evolution of the silkworm adapted to host plant mulberry, many mechanisms have been selected. COEs can be induced by multiple allelochemicals, such as phenolic glycosides, quercetin, rutin. Indeed, mulberry leaves contain these secondary metabolites [44]. The fact that a large numbers of *BmCOEs *were predominantly expressed in the midgut may suggest that these genes might also play important roles in tolerating the relevant allelochemicals. Like the COEs, UDP-glucosyltransferases were also expanded in the silkworm relative to Dipteran and Hymenopteran insects [45]. Thus, these expanded superfamilies may represent the characteristic of phytophagous insect.

Some of midgut carboxylesterases were expressed not only in the midgut, but also in the other tissues such as malpighian tubule, integument, head, fat body and testis. Especially, one cluster in group (I) including seven COE genes was found to be predominantly expressed in the malpighian tubule. The function of insect malpighian tubule is similar to mammalian kidney, which plays important roles in defending against insecticides such as DDT and metabolism of plant secondary and other molecules [46]. Thus, these COEs predominantly expressed in the malpighian tubule may be important detoxification enzymes that eliminate insecticides and allelochemicals. Simultaneously, these midgut carboxylesterases expressed in other tissues had important roles in protecting silkworms from xenobiotic damages.

#### Head and integument-specific expression genes

The head and integument specific group (II) included α-esterase, jhe, β-esterase, integument esterase, and neuroligin genes. Except for the common expression characteristic, these genes were also expressed in other tissues. For example, *Bmae32 *and *Bmnlg6 *showed male-predominant expression in the reproductive system, whereas *Bmun1 *was a female-predominant expression gene. *Bmie1 *and *Bmie2 *were also expressed in homocyte, and the former showed higher expression level in the anterior/median silk gland (A/MSG) and the latter was also expressed in fat body, midgut, and malpighian tubule.

**Figure 5:**
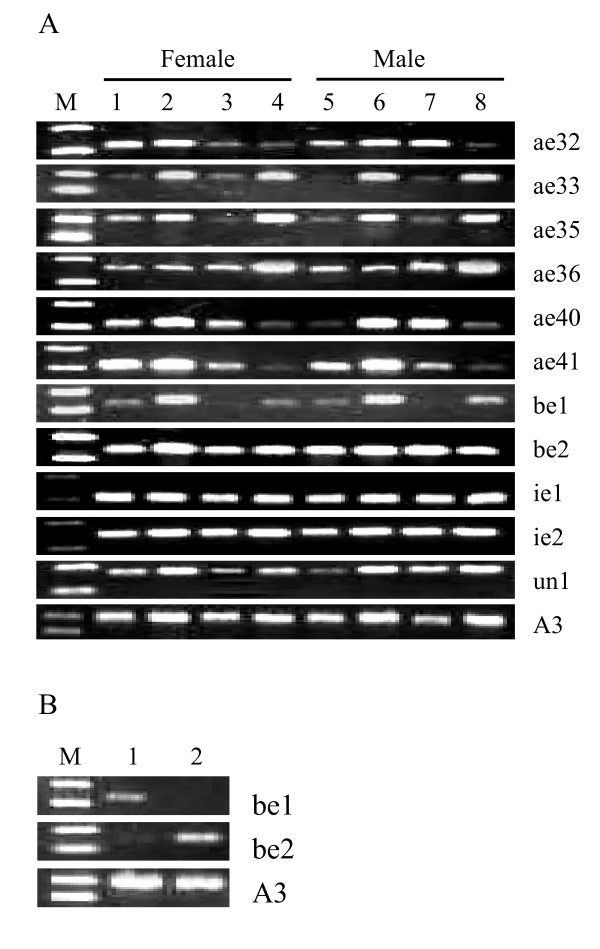
**Expression patterns of the silkworm COEs validated by RT-PCR**. (a) Expression of the silkworm COEs in special tissues. M: DL2000 marker, 1 and 5: larvae antennae, 2 and 6: larvae maxilla, 3 and 7: adult antennae, 4 and 8: adult leg. (b) Expression of β-esterases in adult accessory gland. M: DL2000 marker, 1: male accessory gland, 2: female accessory gland.

ODEs/PDEs, antennal and integument esterases play important roles in degrading odorants/pheromones, such as volatile acetate compounds; these enzymes belong to α-esterase, β-esterase and integument esterase [7,29]. In addition, for the herbivorous insects, odorant/pheromone-degrading esterase may have a role in the degradation of plant volatiles with an ester functional group [47]. Previous studies showed that *Apol*-ODE and *Apol*-PDE could be detected in adult male antennae, but not in female antennae and other control tissues [36]. *Apol*-IE, *Mbra*-EST, *Slit*-EST and *Snon*-EST were expressed in male and female antennae whereas *Slit*-EST and *Snon*-EST showed higher expression in legs [28,29,47]. For the silkworm, only larvae fetch mulberry leaves and also encounter a mass of volatile allelochemicals, such as hexyenyl acetate, 3-hexenyl acetate and 2-hexenyl acetate [48]. Based on the microarray data, it was found that *BmCOEs *phylogenetically related to odorant/pheromone-degrading esterases were specifically expressed in the head and integument of the silkworm larvae (Figure 4, group II). Thus, we employed RT-PCR technique to analyze the expression patterns in larva antenna and maxilla, on which important olfactory sensilla are distributed [48], and adult antennae and leg to presume the function of head and integument-specific expression esterases, excluding *Bmnlg6 *and *Bmjhe1*. The results indicated that most of the head and integument-specific expression esterases were expressed in larva antennae and maxilla (Figure 5a). Except for the *Bmae35, ae40 *and *un1*, others did not show obvious expression differences between female and male. The antenna and maxilla of the silkworm larvae are important olfactory organs. Thus, we predicted that these esterases would play important roles in degrading the volatile acetate allelochemicals and adaptive evolution of silkworm with mulberry leaves. In addition, The pheromone of *B. mori *is a blend of alcohol (bombykol) and aldehyde (bombykal) [49]. Antennal specific aldehyde oxidase (AOX) can degrade bombykal [7]. Bombykol was inactivated firstly by conversion, including the oxidation of the pheromones by oxidases and/or dehydrogenases to the corresponding fatty acids, and secondly by degradation [50]. Thus, odorant/pheromone-degrading like esterases in the silkworm may indirectly play role in degrading the pheromones. Furthermore, *Bmae33*, *ae35 *and *Bmbe1 *showed lower or no expression in adult antennae, suggesting that they may have different functions from their corresponding orthologous genes. In addition, it was observed that some head and integument-specific esterases were also expressed in adult antennae and legs. So, the functions of these esterases expressed both in the silkworm larvae and adult moths need to be further determined.

#### Silk gland-specific expression genes

In the silk gland specific group (III), nine genes were found, including α-esterase and JHE (Figure 4). *Bmae11, ae43*, and *ae50 *were only expressed in the silk gland, especially *Bmae50 *only in the anterior/median silk gland. However, Bmjhe4 was also expressed in the head of females, Bmae25 in the head of females and fat body in males, and Bmae23 in the head and midgut in females/males, and fat body in males. Silkworm is an economically important insect, which can efficiently synthesize silk proteins. Silk gland in the silkworm is an organ specialized for the synthesis and secretion of silk proteins. Due to the fact that COEs have the activities of hydrolase and ethyl ester synthase [51], we speculated that silk-gland specifically expressed COEs may participate in synthesis of silk proteins and detoxify the xenobiotics entered into silk gland.

#### Other specific expression genes

In adult, the male accessory glands (MAGs) of many insect species can produce and secrete a number of reproductive proteins that are expressed exclusively or abundantly in the MAGs [52]. The previous studies indicated that *EST-6 *(CG6917) in *Drosophila *is expressed in the male genitalia and transferred to the female during mating, influencing egg-laying behavior and possibly receptivity to remating [53]. In *An. gambiae*, *Agbe1d *and *be4d *homologous to *Drosophila EST-6 *were also specifically expressed in the MAGs [52]. The expression patterns of two silkworm β-esterases in adult accessory glands were investigated. The results indicated that *Bmbe1 *and *Bmbe2 *were specifically expressed in the female accessory glands and MAGs, respectively (Figure 5b). Thus, *Bmbe2 *may have similar function to *Drosophila EST-6*. In addition, silkworm *Bmae10*, *ae15*, *ae30*, *ae32*, and *ae53 *and *Bmnlg3 *and *nlg6 *were expressed in male testis, but not in ovary. We supposed that these differential expression genes in reproductive system may play important roles in spermatogenesis or detoxification to avoid damage of xenobiotics.

*Bmjhe1 *was expressed in the anterior/middle/posterior silk glands in the 4th larval instar and on day 10 in the 5th larval instar just before pupation [33]. In addition, a previous study detected the expression of *Bmjhe1 *only at day 0 in the 5th instar in the fat body of seven tissues [54]. In this study, we observed that *Bmjhe1 *was expressed in midgut, ovary, fat body, and malpighian tubule, and the highest expression level in fat body at day 3 in the 5th instar. Thus, expression of carboxylesterase genes showed not only tissue but also developmental stage specificities.

## Conclusion

A comprehensive search was conducted for potential COE genes in the silkworm genome. *B. mori *contains 76 COE genes, the largest number of COE genes among insects investigated. Relative to Dipteran and Hymenopteran insects, silkworm has experienced a significant expansion for α-esterases. The expanded α-esterases were predominantly expressed in midgut, head and integument, and silkgland, respectively, suggesting that they may participate in allelochemical tolerance and synthesis of silk proteins. Generally, α-esterases contain the conserved catalytic triad and showed catalytic function. However, 15 of 55 silkworm α-esterases mutated at the essential catalytic residue sites, implying that they may acquire some new functions. On the basis of tissue microarray, the putative odorant/pheromone-degrading esterases and related genes predominantly expressed in head and integument of the silkworm larvae were detected. RT-PCR verified that these genes were also expressed in the larvae antenna and maxilla, suggesting that they play important roles in detoxifying plant volatiles. In addition, *Bmbe2 *were specifically expressed in the adult MAGs, which may have a similar function of *Drosophila EST-6*, influencing egg-laying behavior and possibly receptivity to remating. In sum, our results provide some new insight into annotation and evolutionary characteristics of the silkworm COEs.

## Methods

### Identification of the *B. mori *COE genes

COEs amino acid sequences of *D. melanogaster*, *An. gambiae*, and *Ap. mellifera *were downloaded from the GenBank http://www.ncbi.nlm.nih.gov/. We searched the silkworm genome for candidate COEs genes using the tblastn program with the silkworm 9×genome database [19]. Genomic sequences that show even weak sequence similarity to any query sequence and its flanking regions (1 kb or more long) were extracted. Putative COE genes within the extracted sequences were predicted using BGF software [55] and Fgenesh+ http://www.softberry.com/.

### Phylogenetic analysis

Multiple sequence alignments of COEs amino acids were aligned using Clustal X [56]. Positions that have a high percentage of gaps (>70%) were manually trimmed. Phylogenetic tree was reconstructed using the neighbor-joining method in which distance was estimated by JTT amino acid matrix implemented in MEGA 4.0 program [57]. The pairwise deletion option was used in the NJ tree reconstruction and the accuracy of the tree topology was assessed by bootstrap analysis with 100 resampling replicates.

#### Expression analysis with ESTs and microarray data

The putative coding sequences of *BmCOEs *were used as queries to perform Blastn searches against the silkworm EST database downloaded from GenBank. A 95% or greater identity and minimum cut-off E-value (≤e-20) were employed to discriminate between duplicate genes.

A genome-wide 69-mer oligonucleotides microarray with 22,987 probes has previously been customized for the silkworm [24]. Sixty-three of the 76 *BmCOEs *identified in this study were found to have probes on the microarray. The expression patterns of these genes have been surveyed for the 9 representative sample types of Chinese silkworm strains (Dazao) on day 3 of the fifth instar, including silk gland, testis, ovary, fat body, midgut, integument, hemocyte and malpighian tubule, and head. The detailed experimental process, quality control, consistency in replication and data analysis for these experiments have been described in previous report [24].

### Gene expression by reverse transcriptase-polymerase chain reaction (RT-PCR)

Each tissue was dissected and stored in liquid nitrogen before pulverizing. Total RNA was isolated using Trizol Reagent (Invitrogen) according to the manufacturer's instructions. The contaminating genomic DNA was digested with Rnase-free Dnase I (Promega) for 15 min at 37°C. The RNA samples were eluted with Rnase-free water and stored at -80°C. The first strand of cDNA was synthesized using M-MLV reverse transcriptase following the manufacturer's instructions (Promega, USA).

RT-PCR primers were designed on the basis of the coding sequences of the silkworm COEs (Additional file 5). The silkworm cytoplasmic actin (*A3*) gene (accession No. U49854) was used as an internal control. PCR amplification reactions were performed in 25 μl volumes containing normalized cDNA, 0.2 mM of each primer, 2 mM MgCl_2_, 0.25 mM dNTP, 1× buffer and 2.5 units of Taq DNA polymerase (Promega). The PCR cycling program had an initial denaturation step of 95°C for 4 min, followed by 25 cycles of 94°C for 30 s, 30 s annealing (temperatures listed in Additional file 5), 40 s extension (72°C), and a final extension at 72°C for 10 min. The amplification products were analyzed on 1.2% agarose gels.

## Authors' contributions

QYY and CL made the study design. QYY did the data collection and analysis, and drafted the manuscript. WLL did partial data analysis. ZZ revised the manuscript. ZZ and ZHX supervised the study. All authors read and approved the final manuscript.

## Supplementary Material

Additional file 1 **Summary of the silkworm COE genes**. NP indicates genes with no automatic prediction in silkworm. (N): missing N-terminal region; (C): missing C-terminal region. Chr.: chromosome. UN represents unknown chromosome locations. Click here for file

Additional file 2 **Sequence alignment of Lepidopteran AChEs**. Three intrachain disulfide bridges are drawn between conserved Cys. The asterisks represent the catalytic triad (S200, E327 and H440). Click here for file

Additional file 3 **Chromosome distribution of all silkworm COEs**. Arrows showed the transcriptional orientation. Click here for file

Additional file 4 **The phylogeny and expression patterns of *BmCOEs***. Bootstrap values > 70% are shown. The solid black circles corresponding to cDNA, EST, tissue microarray and RT-PCR column indicate that these genes have expression evidence. The genes with grey font are that they had no probes in microarray dataset. The underlined α-esterases mean that they had substitutions in the catalytic triad. Click here for file

Additional file 5 **Primers used in RT-PCR study**. Primers used in RT-PCR study. Click here for file
