# Supplementary material for: Annotation and expression of carboxylesterases in the silkworm, Bombyx mori
Source: BMC Genomics. 2009 Nov 24;10:553. doi: 10.1186/1471-2164-10-553 (PMC2784812; doi:10.1186/1471-2164-10-553)
Supplement: Additional file 1 — Summary of the silkworm COE genes. NP indicates genes with no automatic prediction in silkworm. (N): missing N-terminal region; (C): missing C-terminal region. Chr.: chromosome. UN represents unknown chromosome locations. [file 1471-2164-10-553-S1.DOC]

| Gene  name | Accession no. | Length of protein | Chr. | scaffold | EST | Microarray  probe no. |
| --- | --- | --- | --- | --- | --- | --- |
| Bmae1 | BGIBMGA000729 | 538 | 1 | nscaf1690 | - | sw01246 |
| Bmae2 | BGIBMGA006081 | 541 | 4 | nscaf2847 | + | sw12242 |
| Bmnlg1 | BGIBMGA006082 | 648 (C) | 4 | nscaf2847 | - |  |
| Bmnlg2 | BGIBMGA006202 | 935 | 4 | nscaf2847 | - | sw03792 |
| Bmae3 | EU688969 | 650 | 5 | nscaf2529 | + | sw15605 |
| Bmae4 | BGIBMGA003517 | 540 | 5 | nscaf2674 | - | sw09185 |
| Bmnrt1 | BGIBMGA003724 | 416 (C) | 5 | nscaf2674 | + |  |
| Bmgli | BGIBMGA005805 | 854 | 5 | nscaf2838 | + | sw19669 |
| Bmun1 | DQ443247 | 691 | 6 | nscaf2853 | + | sw00733 |
| Bmbe1 | BGIBMGA009874 | 558 | 8 | nscaf2970 | + | sw04565 |
| Bmace2 | AB161180 | 638 | 9 | nscaf3045  /nscaf3047 | + | sw04967 |
| Bmae5 | BGIBMGA002796 | 534 | 10 | nscaf2575 | + | sw20245 |
| Bmae6 | BGIBMGA002899 | 475 (C) | 10 | nscaf2575 | - |  |
| Bmae7 | BGIBMGA002900 | 533 | 10 | nscaf2575 | - |  |
| Bmae8 | BGIBMGA002901 | 534 | 10 | nscaf2575 | - |  |
| Bmae9 | BGIBMGA002902 | 533 | 10 | nscaf2575 | + |  |
| Bmae10 | BGIBMGA001629 | 530 | 11 | nscaf2176 | - | sw09787 |
| Bmae11 | BGIBMGA001630 | 532 | 11 | nscaf2176 | - | sw15854 |
| Bmae12 | BGIBMGA011893 | 564 | 11 | nscaf3032 | + | sw01214 |
| Bmae13 | DQ311250 | 540 | 11 | nscaf3034 | + | sw12117 |
| Bmae14 | BGIBMGA012122 | 555 | 11 | nscaf3034 | + | sw21154 |
| Bmae15 | BGIBMGA010328 | 478 (C) | 12 | nscaf2990 | - | sw08854 |
| Bmae16 | BGIBMGA010505 | 590 | 12 | nscaf2993 | + | sw12821 |
| Bmae17 | BGIBMGA000837 | 542 | 13 | nscaf1898 | + | sw21466 |
| Bmae18 | BGIBMGA000875 | 531 | 13 | nscaf1898 | - | sw07946 |
| Bmnrt2 | BGIBMGA001242 | 723 (N, C) | 13 | nscaf1898 | + | sw11290 |
| Bmae19 | EU523532 | 545 | 13 | nscaf3093 | + | sw13684 |
| Bmae20 | BGIBMGA009337 | 476 (C) | 14 | nscaf2948 | - | sw19383 |
| Bmnlg3 | BGIBMGA002170 | 900 (N, C) | 15 | nscaf2216 | + | sw14720 |
| Bmnlg4 | BGIBMGA002173 | 927 | 15 | nscaf2216 | - | sw02454 |
| Bmnlg5 | BGIBMGA002180 | 982 | 15 | nscaf2216 | - | sw16337 |
| Bmnlg6 | BGIBMGA002185 | 894 | 15 | nscaf2216 | - | sw12843 |
| Bmace1 | AB189740 | 683 | 15 | nscaf2655 | + | sw01801 |
| Bmae21 | NP | 567 | 15 | nscaf2887 | + | sw11948 |
| Bmae22 | BGIBMGA007545 | 566 | 15 | nscaf2887 | - | sw11949 |
| Bmae23 | BGIBMGA007546 | 550 | 15 | nscaf2887 | + | sw06448 |
| Bmae24 | BGIBMGA007547 | 555 | 15 | nscaf2887 | + | sw14575 |
| Bmae25 | EU523534 | 559 | 15 | nscaf2888 | + | sw22280 |
| Bmae26 | BGIBMGA007671 | 573 | 15 | nscaf2888 | + | sw06462 |
| Bmae27 | BGIBMGA001841 | 559 | 19 | nscaf2204 | + | sw03697 |
| Bmae28 | BGIBMGA001856 | 438 (N) | 19 | nscaf2204 | - | sw20556 |
| Bmae29 | BGIBMGA001964 | 580 | 19 | nscaf2204 | + |  |
| Bmae30 | NP | 614 | 19 | nscaf2204 | - | sw15265 |
| Bmae31 | BGIBMGA004147 | 549 | 19 | nscaf2770 | + |  |
| Bmae32 | BGIBMGA004205 | 540 | 19 | nscaf2770 | - | sw13499 |
| Bmae33 | BGIBMGA004206 | 554 | 19 | nscaf2770 | + | sw09501 |
| Bmae34 | BGIBMGA004207 | 445 (N, C) | 19 | nscaf2770 | - | sw01590 |
| Bmbe2 | EU727141 | 572 | 19 | nscaf3052 | + | sw01051 |
| Bmae35 | BGIBMGA004229 | 526 | 20 | nscaf2780 | + | sw06237 |
| Bmae36 | BGIBMGA004683 | 538 | 22 | nscaf2811 | + | sw16332 |
| Bmae37 | BGIBMGA004684 | 539 | 22 | nscaf2811 | + |  |
| Bmae38 | BGIBMGA012722 | 469 (C) | 22 | nscaf3056 | + | sw19995 |
| Bmae39 | NP | 506 | 22 | nscaf3056 | + | sw20808 |
| Bmae40 | EU523535 | 545 | 22 | nscaf3056 | + | sw09098 |
| Bmae41 | EU727142 | 541 | 22 | nscaf3056 | + | sw06296 |
| Bmae42 | BGIBMGA010969 | 519 | 23 | nscaf3013 | - | sw11950 |
| Bmae43 | BGIBMGA010976 | 552 | 23 | nscaf3013 | + | sw07726 |
| Bmae44 | BGIBMGA010986 | 538 | 23 | nscaf3013 | - |  |
| Bmae45 | EU255790 | 535 | 23 | nscaf3013 | + |  |
| Bmae46 | BGIBMGA010988 | 531 | 23 | nscaf3013 | - | sw07727 |
| Bmae47 | EF017799 | 640 | 23 | nscaf3026 | + |  |
| Bmae48 | DQ443360 | 756 | 23 | nscaf3026 | + | sw05730 |
| Bmae49 | EU688968 | 550 | 24 | nscaf2891 | + | sw05060 |
| Bmjhe1 | AF287267 | 570 | 25 | nscaf1705 | + | sw14035 |
| Bmae50 | BGIBMGA000773 | 503 (N) | 25 | nscaf1705 | - | sw08757 |
| Bmjhe2 | BGIBMGA000774 | 566 | 25 | nscaf1705 | - | sw00495 |
| Bmjhe3 | BGIBMGA000775 | 567 (N) | 25 | nscaf1705 | - | sw03964 |
| Bmjhe4 | BGIBMGA000776 | 572 | 25 | nscaf1705 | + | sw03795 |
| Bmae51 | BGIBMGA000777 | 456 (N,C) | 25 | nscaf1705 | + |  |
| Bmae52 | NP | 484 (N,C) | 25 | nscaf1705 | - | sw13618 |
| Bmae53 | BGIBMGA004870 | 604 | 25 | nscaf2818 | + | Sw05012 |
| Bmae54 | BGIBMGA004934 | 568 | 25 | nscaf2822 | + | sw16255 |
| Bmie1 | EU328352 | 561 | 28 | nscaf3098 | + | sw20886 |
| Bmae55 | BGIBMGA014599 | 567 | UN | scaffold914 | - | sw22224 |
| Bmie2 | EU647213 | 571 | UN | nscaf2954 | + | sw08442 |
| Bmun2 | BGIBMGA014463 | 485 (N) | UN | scaffold700 | - | sw13297 |
