# Supplementary figures and images for: Annotation and expression of carboxylesterases in the silkworm, Bombyx mori
Source: BMC Genomics. 2009 Nov 24;10:553. doi: 10.1186/1471-2164-10-553 (PMC2784812; doi:10.1186/1471-2164-10-553)

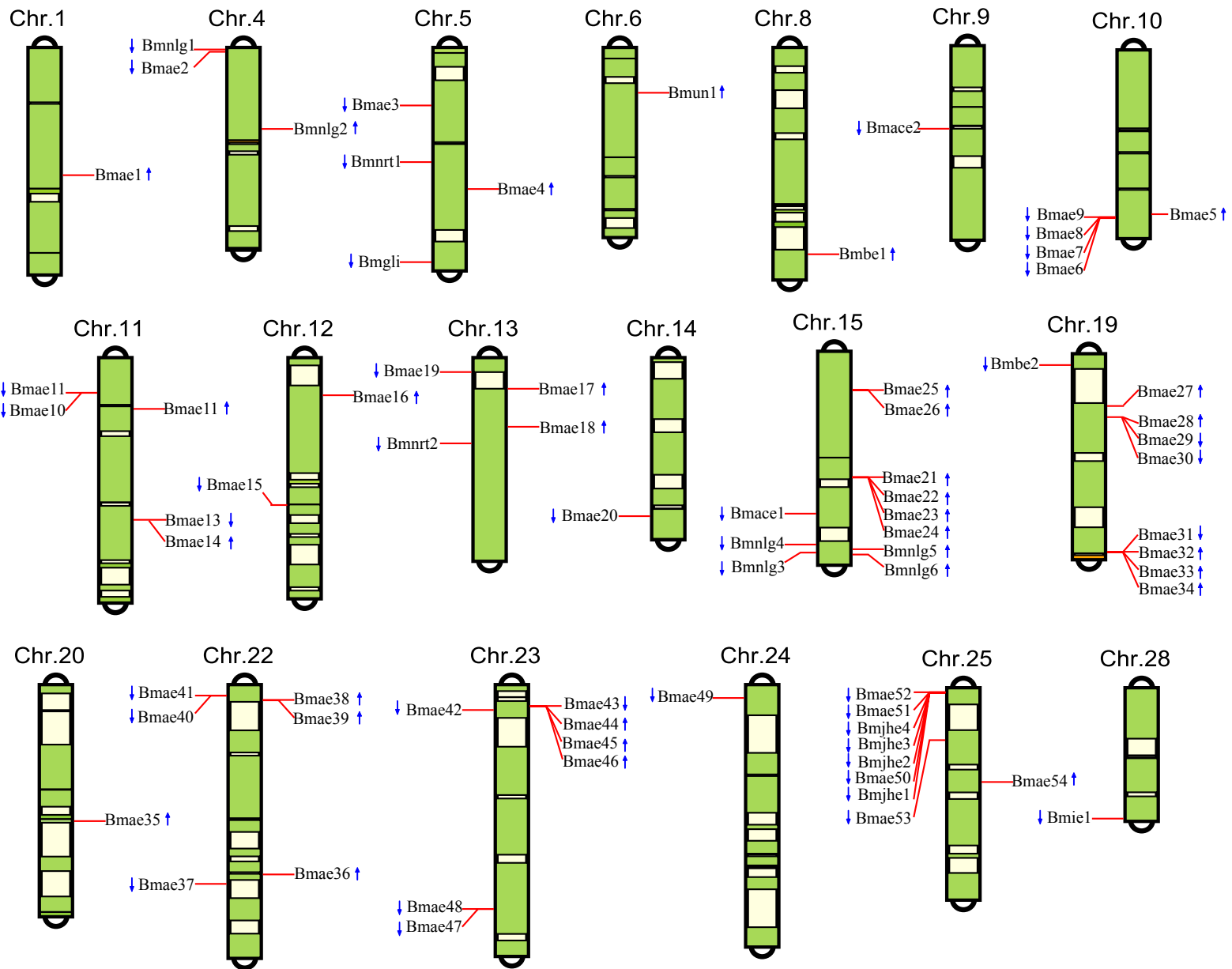

Supplement: Additional file 3 — Chromosome distribution of all silkworm COEs. Arrows showed the transcriptional orientation. [file 1471-2164-10-553-S3.PDF]
