# Supplementary material for: Annotation and expression of carboxylesterases in the silkworm, Bombyx mori
Source: BMC Genomics. 2009 Nov 24;10:553. doi: 10.1186/1471-2164-10-553 (PMC2784812; doi:10.1186/1471-2164-10-553)
Supplement: Additional file 5 — Primers used in RT-PCR study. Primers used in RT-PCR study. [file 1471-2164-10-553-S5.DOC]

| Gene name | Forward primer (5'-3') | Reverse primer (5'-3') | Length of  amplicon (bp) | Annealing tem-  Peratures (℃) |
| --- | --- | --- | --- | --- |
| *Bmae32* | TACCATACGCCGCAACTGAC | CTGAGCCATCTCGGAACCCT | 237 | 56 |
| *Bmae33* | TGGTAATACGGACAAAGAGGG | GCAACGGAAAGGAGTGAGG | 416 | 52 |
| *Bmae35* | GCAAACATCTCACCCAACACG | GGCTCCTCCTCCGTAACTCTG | 212 | 56 |
| *Bmae36* | ACCCACAACCTTCCTATCTCG | GCCGTTGTAGTAAATATAGAAGAGG | 335 | 51 |
| *Bmae40* | TAAAGACGCTCCAGGAAACGC | CGCCCAACTAGATATTGCAGAAC | 220 | 57 |
| *Bmae41* | TCCTGACAATGTTACCCTCTT | TCTTTATCATTCGAGCCGTAG | 423 | 50 |
| *Bmbe1* | TCAGGAAACATACCCGCAACT | AACCTAAGCTATCCCAGAACG | 455 | 53 |
| *Bmbe2* | CGGTAACAATGGTCTGAAAG | CGATAGCATGTGGAAGTGA | 142 | 50 |
| *Bmie1* | TGCCGCACATCATCAGCC | CGTAGATGGTGTCGTCGTGGT | 408 | 55 |
| *Bmie2* | GTGTTATTGTTTGCTGCTTATGG | TAGTTCGGATTAGTTGGTTGC | 305 | 51 |
| *Bmun1* | CGCCACGAGACAATGCTTACC | TCCATACGCAGACGCAGAACC | 187 | 58 |
| *Bmactin3* | AACACCCCGTCCTGCTCACTG | GGGCGAGACGTGTGATTTCCT | 666 | 55 |
