# Supplementary material for: Annotation and expression of carboxylesterases in the silkworm, Bombyx mori
Source: BMC Genomics. 2009 Nov 24;10:553. doi: 10.1186/1471-2164-10-553 (PMC2784812; doi:10.1186/1471-2164-10-553)
Supplement: Additional file 2 — Sequence alignment of Lepidopteran AChEs. Three intrachain disulfide bridges are drawn between conserved Cys. The asterisks represent the catalytic triad (S200, E327 and H440). [file 1471-2164-10-553-S2.PDF]

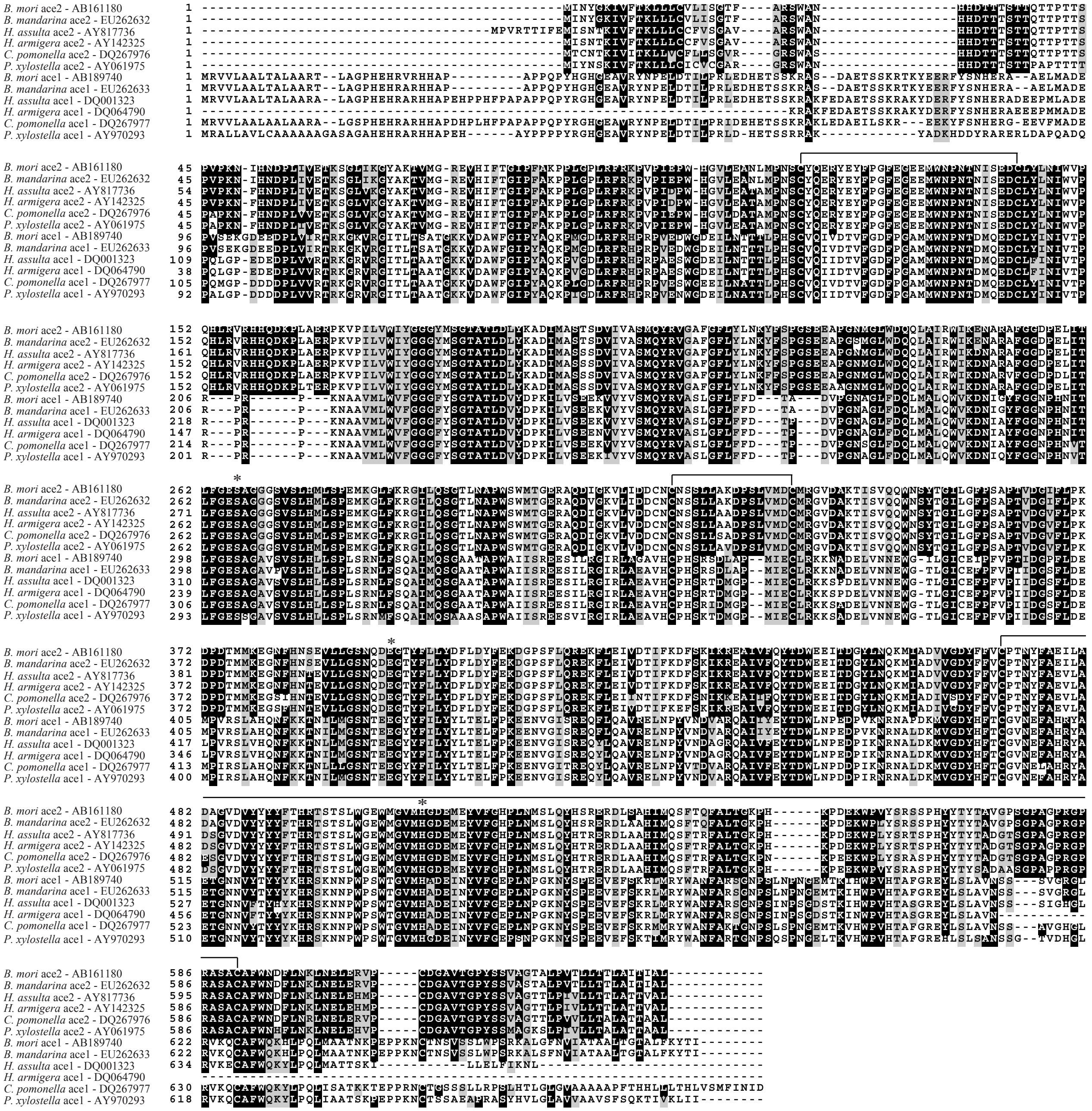
B. mori ace2 - AB161180
B. mandarina ace2 - EU262632
H. assulta ace2 - AY817736
H. armigera ace2 - AY142325
C. pomonella ace2 - DQ267976
P. xylostella ace2 - AY061975
B. mori ace1 - AB189740
B. mandarina ace1 - EU262633
H. assulta ace1 - DQ001323
H. armigera ace1 - DQ064790
C. pomonella ace1 - DQ267977
P. xylostella ace1 - AY970293
