# Supplementary material for: Annotation and expression of carboxylesterases in the silkworm, Bombyx mori
Source: BMC Genomics. 2009 Nov 24;10:553. doi: 10.1186/1471-2164-10-553 (PMC2784812; doi:10.1186/1471-2164-10-553)
Supplement: Supplementary file 5 [file 1471-2164-10-553-S4.DOC]

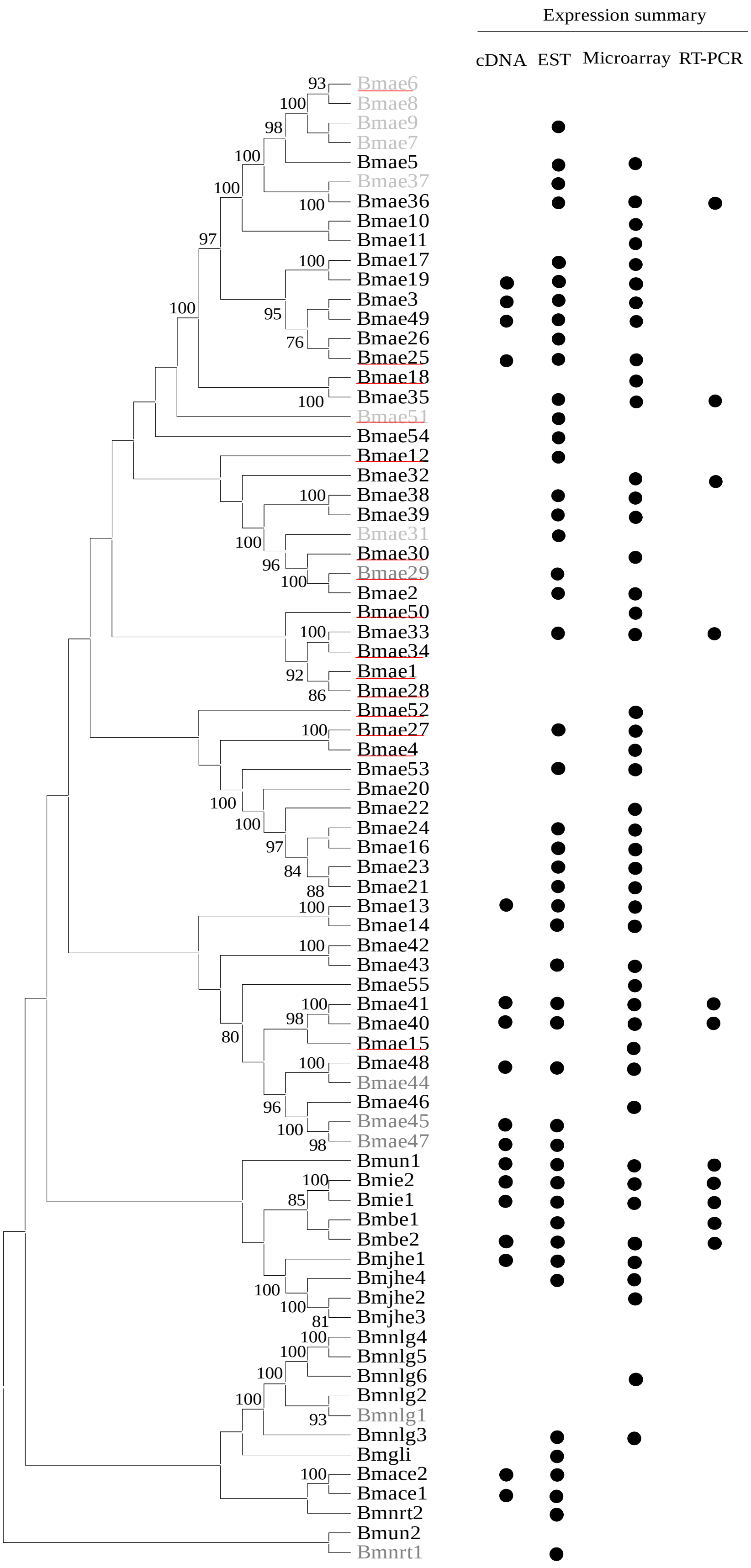

Expression summary
cDNA
EST
Microarray
RT-PCR
Bmae6
Bmae8
Bmae9
Bmae7
Bmae5
Bmae37
Bmae36
Bmae10
Bmae11
Bmae17
Bmae19
Bmae3
Bmae49
Bmae26
Bmae25
Bmae18
Bmae35
Bmae51
Bmae54
Bmae12
Bmae32
Bmae38
Bmae39
Bmae31
Bmae30
Bmae29
Bmae2
Bmae50
Bmae33
Bmae34
Bmae1
Bmae28
Bmae52
Bmae27
Bmae4
Bmae53
Bmae20
Bmae22
Bmae24
Bmae16
Bmae23
Bmae21
Bmae13
Bmae14
Bmae42
Bmae43
Bmae55
Bmae41
Bmae40
Bmae15
Bmae48
Bmae44
Bmae46
Bmae45
Bmae47
Bmun1
Bmie2
Bmie1
Bmbe1
Bmbe2
Bmjhe1
Bmjhe4
Bmjhe2
Bmjhe3
Bmnlg4
Bmnlg5
Bmnlg6
Bmnlg2
Bmnlg1
Bmnlg3
Bmgli
Bmace2
Bmace1
Bmnrt2
Bmun2
Bmnrt1
